# Draft Genome Sequence of *Aeromonas hydrophila* Strain BSK-10 (Serotype O97), Isolated from *Carassius carassius* with Motile Aeromonad Septicemia in China

**DOI:** 10.1128/genomeA.00497-17

**Published:** 2017-06-15

**Authors:** Xiaoyi Pan, Lingyun Lin, Yang Xu, Xuemei Yuan, Jiayun Yao, Wenlin Yin, Guijie Hao, Jinyu Shen

**Affiliations:** Agriculture Ministry Key Laboratory of Healthy Freshwater Aquaculture, Zhejiang Institute of Freshwater Fisheries, Huzhou, Zhejiang, People's Republic of China

## Abstract

We report here a draft genome sequence of *Aeromonas hydrophila* strain BSK-10, belonging to serotype O97, isolated from crucian carp (*Carassius carassius*) with motile aeromonad septicemia in Zhejiang, China. The assembly resulted in 34 scaffolds totaling approximately 4.97 Mb, with an average G+C content of 60.97% and 4,594 predicted coding genes.

## GENOME ANNOUNCEMENT

*Aeromonas hydrophila* is a Gram-negative, rod-shaped bacterium that is ubiquitous in aquatic environments (1). Being an opportunistic pathogen, *A. hydrophila* often causes disease in fish, amphibians, reptiles, and mammals (2). Motile aeromonad septicemia (MAS), which is caused by mesophilic *A. hydrophila*, has been a severe infectious disease in cultivated freshwater fish throughout China since 1989 (3, 4). Hundreds of mesophilic *A. hydrophila* strains have been isolated from diseased fish, including crucian carp, silver carp, bighead carp, and blunt-snout bream (5, 6). The *Aeromonas* genus includes a total of 97 serotypes, but only some of them, such as O3, O6, O9, O11, O14, O16, O18, O21, O29, O33, O34, O41, and O97, seem to be associated with virulence for specific fish species (3, 7). The main Chinese epidemic strains of *A. hydrophila* were serotyped using the National Institute of Health (Japan) serotyping system of Sakazaki and Shimada (8, 9). Serotyping revealed two dominant serotypes (O9 and O97) among *A. hydrophila* isolates in China (3, 6). Here, we report the draft genome sequence of strain BSK-10 belonging to serotype O97, which was isolated in 1990 from kidney tissue of a moribund crucian carp (*Carassius carassius*) with MAS in Huzhou city, Zhejiang province, China (5).

The *A. hydrophila* BSK-10 genome was sequenced using the Illumina Hiseq2000 platform at Meiji Biotech (Shanghai, China), which generated a total of 10,339,809 paired reads with an average coverage of 415.69×. Adaptor trimming and quality control of the sequence reads were conducted using CLC Genomics Workbench version 10.0.1 (CLC bio). *De novo* assembly was performed with CLC Genomics Workbench version 10.0.1, and then the contigs were joined based on reads, paired reads, and overlaps between contigs with the CLC Genomics Workbench genome finishing module. The scaffolds were annotated using the NCBI’s Prokaryotic Genome Annotation Pipeline (10).

*De novo* assembly generated 152 contigs, and the resulting *N*_50_ size of the contigs was 368.028 kb. The draft genome, comprising 34 scaffolds, contained 4,963,306 bp with an average G+C content of 60.97%, which is consistent with the other *A. hydrophila* strains previously sequenced, namely, ATCC 7966 (61.5%), ML09-119 (60.8%), and J-1 (60.9%) (11–13). The draft genome of BSK-10 contained 4,594 genes encoding 4,482 open reading frames, 98 tRNAs, 7 rRNAs, 6 ncRNAs, and 1 tmRNA. According to the results of the annotation, we found five secretion systems in the BSK-10 genomes, namely, type I, II, III, IV, and VI secretion systems. In addition, 36 genomic islands (GIs) were predicted by IslandViewer version 3 software (14). By analyzing the gene content of the 36 GIs, we found one O-antigen gene cluster with 33,847 bp and 29 genes. Moreover, genomic analysis revealed that *A. hydrophila* BSK-10 contains genes encoding previously described virulence-associated factors, such as adhesins, enterotoxin, RTX toxin, hemolysins, lipases, type IV pilus, and proteases.

### Accession number(s).

The draft genome sequence of *A. hydrophila* BSK-10 has been deposited at DDBJ/ENA/GenBank under the accession number NBOV00000000. The version described in this paper is the first version, NBOV01000000.
